# Identification of Differentially Expressed Genes and Pathways Involved in Growth and Development of *Mesona chinensis* Benth Under Red- and Blue-Light Conditions

**DOI:** 10.3389/fpls.2021.761068

**Published:** 2021-11-25

**Authors:** Danfeng Tang, Qinfen Huang, Kunhua Wei, Xiaonan Yang, Fan Wei, Jianhua Miao

**Affiliations:** ^1^Guangxi Key Laboratory of Medicinal Resources Protection and Genetic Improvement, Guangxi Botanical Garden of Medicinal Plants, Nanning, China; ^2^Guangxi Engineering Research Center of TCM Resource Intelligent Creation, Guangxi Botanical Garden of Medicinal Plants, Nanning, China

**Keywords:** *Mesona chinensis* Benth, plant factory, LED, red and blue light, growth and development

## Abstract

*Mesona chinensis* Benth (MCB) is an important Chinese herbal medicine. The plant factories might be one of the ways to solve the shortage of MCB supply. In this study, the MCB seedlings were treated under the red (R) and blue (B) lights in the plant factory. Results showed that the red light promoted the growth and development of MCB in comparison with the blue light. Under the red-light condition, the biomass, plant height, and root characteristics were significantly higher than those under blue-light condition, while the soil and plant analyzer development (SPAD) under the red-light treatment was significantly lower than that under the blue-light treatment. Red light also significantly promoted the content of soluble sugar and pectin of MCB compared with blue light. Transcriptome analysis showed that a total of 4,165 differentially expressed genes (DEGs) were detected including 2,034 upregulated and 2,131 downregulated. Of these, 1,112 DEGs including 410 upregulated and 702 downregulated genes were associated with 111 pathways. Moreover, a total of 8,723 differentially expressed transcription factors (TFs) were identified in R vs. B, and these TFs were distributed in 56 gene families. Metabonomic results revealed that a total of 184 metabolites and 99 differentially expressed metabolites (DEMs) (42 upregulated and 57 downregulated) were identified in the red- and blue-light treatments. Integrative analysis of transcriptome and metabolome unveiled that a total of 24 pathways included 70 compounds (metabolites) and were associated with 28 unigenes. In particular, these pathways included starch and sucrose metabolism, phenylpropanoid biosynthesis, cysteine and methionine metabolism, glycolysis/gluconeogenesis, and pentose and glucuronate interconversions. The unigenes included *asparagine synthetase* (*AS*), *thymidine kinase* (*TK*), *alpha, alpha-trehalose-phosphate synthase* (*TPS*), *phosphatase IMPL1* (*IMPL1*), *dihydroflavonol 4-reductase* (*D4R*), and *4-coumarate-CoA ligase-like 6* (*4CL6*), *bifunctional aspartokinase-homoserine dehydrogenase 1* (*thrA*), and *abscisic acid 8′-hydroxylase 2 isoform X1* (*ABA8*). It was indicated that these pathways and genes might play important roles in the growth and development of MCB. This study laid a foundation for the future research of MCB.

## Introduction

*Mesona chinensis* Benth (MCB), belonging to the Lamiaceae family, is an annual or perennial herb. It is an economically important plant widely cultivated in South China and Southeast Asian countries (Ren et al., 2019; Tang et al., 2020). It includes polysaccharides, flavonoids, vitamins, amino acids, fat, fiber, and polyphenols (Su et al., 2011; Tang et al., 2020). *M. Chinensis* Benth polysaccharides (MCP) consist of eight monosaccharides, including galacturonic acid, glucose, galactose, xylose, mannose, rhamnose, ribose, and glucuronic acid, with the molar percentages of 28.4, 26.5, 16.4, 10.6, 7.4, 5.7, 4.2, and 0.9%, respectively (Zhang et al., 2013). As one of the functional active substances, MCP has attracted much attention owing to its various biological activities, including antitumor, antioxidant, antiviral, and hypoglycemic activities (Huang et al., 2018; Wang et al., 2019; Xiao et al., 2019). In addition to its medicinal values, MCB is used as a herbal beverage in China and Southeast Asian countries and also as a source of raw materials in food industries and packaging industries, such as natural food pigment, new refrigerant, food film, and coating agent (Cheng et al., 2015; Yang et al., 2015a,b; Huang et al., 2019; Ren et al., 2019). In recent years, due to a relatively high level of cultivation and management measures of MCB, farmers are not willing to plant it, resulting in the insufficient supply of MCB in China and a large import of MCB raw materials from Southeast Asian countries. Therefore, besides the traditional field cultivation, it is necessary to seek other cultural regimes of MCB.

The plant factory is a revolution for the traditional cropping system to deal with the issues of farmland area shrinkage and population growth (United Nations [UN], 2017). In a plant factory, electric-based equipment is used to control all involved environmental factors, for example, illumination condition, temperature, and nutrition supply (Kim et al., 2013; Zha and Liu, 2018). Light is one of the most important factors that regulate plant growth and development (He et al., 2020a) and that determine photosynthesis and subsequently carbohydrate production and accumulation (Wei et al., 2020). Light-emitting diode (LED) technology provides an essentially distinct and energy-effective approach for the agricultural industries (Ballare et al., 2012). The LED light system allows the regulation of spectrum, spectral composition, and light intensity to supply better growth conditions for commercial crops, fruits, flower plants, and even trees (Yeh and Chung, 2009; Tayebeh et al., 2020). Theoretically, in a plant factory framework, if all the factors are within the most favorable level, some specific plants can grow continuously and efficiently. As mentioned earlier, MCB is an annual or perennial herb and may be suitable for growing in plant factories.

Artificial light is essential in a plant factory, and red (R) and blue (B) lights are the two major wavelengths that drive photosynthesis (Tandeau de Marsac and Houmard, 1993; He et al., 2020c). Red light is a component of the solar spectrum that strongly affects plant tissues (Kuo et al., 2015), while blue light is an important environmental signal for various organisms regulating their growth and developmental processes through photoreceptors (Sano et al., 2009). Although the blue and red lights are essential for the growth of many plants, including potato (He et al., 2020c), watermelon (Bantis et al., 2020), birch (Saebo et al., 1995), lettuce, peanut plants (Poulet et al., 2014; Li et al., 2018), and kidney bean plants (Hiromichi and Kazuhiro, 2000), few studies have focused on the effects of each on the growth of MCB in a plant factory. In this study, we examined and analyzed the physiological, biochemical, cytological, and molecular responses to the red and blue lights in MCB. This study provides guidance for the cultivation of MCB in plant factories and lays a foundation for the future research of MCB molecular biology.

## Materials and Methods

### Materials and Experimental Treatments

*Mesona chinensis* Benth cutting seedlings of about 10–15 cm height were used as plant materials. The seedlings were transplanted on the culture frame in the plant factory with a condition of 25°C room temperature and 70% humidity. The seedlings were exposed to blue (200 μmol m^–2^s^–1^) and red (200 μmol m^–2^s^–1^) lights at a day/night time of 16/8 h, respectively. All the plants were cultivated using the hydroponic method with 1/2 Hoagland nutrient solution. After 1 month, the data on the growth of MCB were measured and collected. Meanwhile, the three-fourth true leaves of apical meristem were collected and frozen at −80°C for the analysis of soluble sugar, soluble pectin, transcriptome, and metabolome (Suzhou PANOMIX Biomedical Tech Co. Ltd., Suzhou, China).

### Determination of Agronomic Characters

Light-emitting diode meter equipment (UPRtek, MK350NPLUS) was used for spectrum measurement. At least three plants from each treatment were taken for the measurement of fresh weight, dry weight, plant height, and soil and plant analyzer development (SPAD) (SPAD-502 Chlorophyll Meter) values. Root morphological indexes were determined using the root analyze system (WinRHIZO, Regent, Canada) (Tang et al., 2019). Soluble sugar and soluble pectin were measured using Plant Soluble Sugar and Pectin Kits (Suzhou Grace Biotechnology Co. Ltd., Suzhou, China).

### Transmission Electron Microscope Observation

The third true leaf of apical meristem was used, and the vein was removed. Transmission Electron Microscope (TEM) observation was referred by Tang et al. (2018). Leaves were cut into small size pieces (about 2 mm × 2 mm) and put into a 2.5% glutaraldehyde buffer solution. Then the samples were fixed at 4°C, rinsed in phosphate buffer, post-fixed in 1% osmium tetraoxide (OsO_4_), dehydrated with a series of 50, 60, 70, 80, 90, and 100% ethanol, washed in 100% acetone, and embedded. Finally, the samples were observed under a TEM system of Hitachi.

### cDNA Library Construction, Sequencing, *de novo* Assembly

The cDNA library was constructed and sequenced according to Santos et al. (2021). Briefly, RNA purity was checked, and RNA integrity was first assessed. Then, about 1 μg RNA per sample was employed for cDNA library construction using NEBNext^®^ Ultra™ RNA Library Prep Kit for Illumina^®^ (NEB, United States), following the instructions of the manufacturer. Consequently, the library quality was estimated using the Agilent Bioanalyzer 2100 system. Finally, the RNA-Seq library sequencing was performed using the Illumina Hiseq X Ten platform for a 150 bp paired-end read.

Trinity^1^ was used for *de novo* assembly of transcriptomes. In brief, clean reads with a certain overlap length were initially combined to form contigs and then related contigs were clustered using the TGICL software (version 2.1) (Pertea et al., 2003) to yield unigenes that could not be extended on either end and redundancies were removed to obtain non-redundant unigenes.

### Functional Annotation of the Assembled Unigenes

The sequences of unigenes were searched against the NR,^2^ KEGG,^3^ GO,^4^ COG,^5^ Swiss-Prot,^6^ and TrEMBL databases (*E*-value ≤ 1E-5) using BLASTX to retrieve protein functional annotations based on sequence similarity. High-priority databases (followed by NR, Swiss-Prot, and KEGG) were selected to determine the direction of the unigene sequences. The best aligning results were used to predict the coding region sequences from unigenes, and the coding sequences (CDSs) were translated into amino sequences using the standard codon table. The ESTScan software (Iseli et al., 1999) was used to decide the sequence direction of the unigenes that could not be aligned to any of the above databases. GO terms were assigned to each sequence annotated using BLASTX against the Nr database using the Blast2GO program with the *E*-value threshold of 1E-5 for further functional categorization. The WEGO software (Ye et al., 2006) was used to plot the distribution of the GO functional classification of the unigenes. The unigene sequences were also aligned to the COG database to predict and classify possible functions and assigned to KEGG pathway annotations to analyze the inner-cell metabolic pathways and the related gene function using BLASTX.

### Differential Expression Analysis and Functional Enrichment

HTSeq was used to calculate the number of reads mapped to each gene and the FPKM (fragments per kilobase of exon model per million mapped fragments) method was employed for the calculation of gene expression. Differential expression analysis was performed using the DgSeq2, *q*-value (or FDR) < 0.01, and | log 2 (fold change [FC])| > 1 was set as the threshold for significantly differential expression. GO enrichment analysis of differentially expressed genes (DEGs) was carried out using the GOseq, in which gene length bias was corrected. GO functional analysis included GO functional classification annotation for DEGs and GO functional enrichment analysis for DEGs (Gene Ontology database, see text footnote 4). The top 10 GO terms with the lowest *p*-value (the most significant enrichment) were selected from each GO category for display. KO-Based Annotation System (KOBAS) was used to test the statistical enrichment of DEGs in KEGG pathways (see text footnote 3). According to the results of DEGs of KEGG enrichment analysis, the top 30 pathways with the lowest *p*-value (the most significant enrichment) were selected for display.

### Liquid Chromatography-Mass Spectrometry Detection

The extraction of metabolites was conducted as follows. All samples were taken in a 2 ml EP tube, two steel balls were added and ground in the tissue grinder at 50 Hz for 60 s, and then the samples were homogenized. Accurately weighed 100 mg (±1%) of the homogenized sample was taken in a 2 ml EP tube, accurately weighed 0.6 ml of methanol (including internal standard) was added, and the mixture was vortexed for 30 s. Two steel balls were added and ground in the tissue grinder for 60 s at 50 Hz. The mixture was centrifuged at 4°C for 10 min at 12,000 rpm, and the supernatant was filtered through 0.22 μm membrane to obtain the prepared samples for the detection of liquid chromatography-mass spectrometry (LC-MS). Of note, 20 μl from each sample was taken to the quality control (QC) samples (samples that were used to monitor deviations of the analytical results from these pool mixtures and compare them with the errors caused by the analytical instrument itself). The rest of the samples were used for the detection of LC-MS according to Zhang et al. (2020).

The raw LC-MS data were converted into mzXML format files by Proteowizard Data Analysis software (version v3.0.8789). Then, peaks identification, peaks filtration, and peaks alignment were processed using XCMS^7^ with the following default set: ppm = 15, bw = 2, peak width = c(5, 30), mzdiff = 0.01, mzwid = 0.015, and method = centWave. Each metabolite was confirmed based on their exact molecular weights (MWs), and the possible empirical formulae of the metabolites were speculated (MW error < 20 ppm). Then, the exact MWs were employed to identify potential biomarkers using Metlin,^8^ Human Metabolome Database (HMDB),^9^ massbank,^10^ mzCloud,^11^ Lipid Maps,^12^ and database built by Bionovogene Co. Ltd.

### Quantitative Reverse Transcription-PCR Analysis

cDNA was synthesized using TransScript^®^ One-Step gDNA Removal and cDNA Synthesis SuperMix, and quantitative reverse transcription-PCR (qRT-PCR) was conducted using PerfectStart^®^ Green qPCR SuperMix (TransGen Biotech Co. Ltd.) on an applied biosystems (Thermo Fisher Scientific). The qPCR primers were designed and listed in Supplementary Table 1. The 20 μl qPCR reaction mixture contained 1.0 μl of cDNA, 0.4 μl of primers, 10 μl of PerfectStart^®^ Green qPCR SuperMix, and 8.2 μl of nuclease-free water. The qPCR amplification procedure was as follows: 94°C for 30 s, followed by 40 cycles of 94°C for 5 s, 60°C for 15 s, and 72°C for 10 s. Each sample was analyzed in triplicate, and the relative gene expression was calculated using the 2^–△△*CT*^ method (Livak and Schmittgen, 2001).

### Statistical Analysis

Means were compared using the least significant differences (Duncan) at the 5% probability level. GraphPad Prism 7, Microsoft Office PowerPoint, and Microsoft Excel were used for data processing and plotting figures.

## Results

### Red Light Promoted the Growth and Development and Quality of *Mesona chinensis* Benth

**FIGURE 1:**
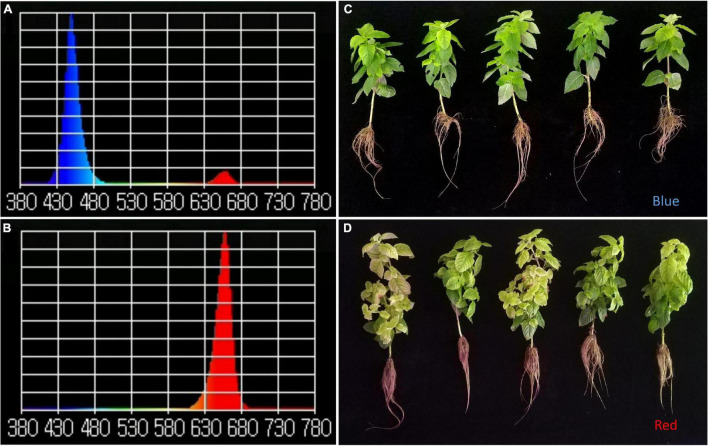
Comparison of plant morphological characteristics and spectrum under the red and blue lights. **(A,B)** Blue- and red-light spectrum, respectively; Y-axis: λpv, X-axis: wavelength; **(C,D)** The plants grown under the blue- and red-light conditions, respectively.

**FIGURE 2:**
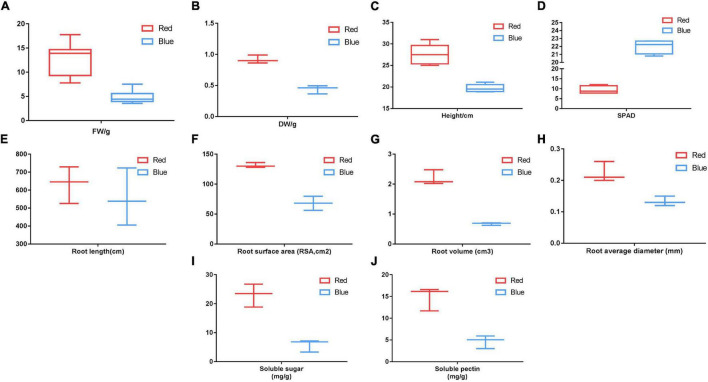
Comparison of plant growth and development, soluble sugar, and soluble pectin under the red and blue lights. **(A–J)** Indiated the fresh weight, dry weight, plant height, SPAD, root length, root surface area, root volume, root average diameter, soluble sugar, and soluble pectin, respectively. FW: fresh weight; DW: dry weight; SPAD, soil and plant analyzer development.

In this study, to ensure the accuracy of the spectrum in the plant factory, the spectra of the red and blue lights were determined (Figures 1A,B). Red light promoted the growth and development of MCB in comparison with blue light (Figures 1C,D). Under the red-light condition, the biomass, plant height, and root characteristics of MCB were significantly higher than those under blue-light condition, while the SPAD of red-light treatment was significantly lower than that of blue-light treatment (Figure 2). Of these, the dry weight, fresh weight, and plant height increased by 96.90, 163.07, and 40.20%, respectively (Figures 2A–C). Compared with blue-light condition, the root length, root surface area, root volume, and root average diameter under red-light condition increased by 13.99, 93.05, 228.22, and 68.25%, respectively (Figures 2E–H). However, under red-light condition, the SPAD value was reduced by 57.50% in comparison with that under blue-light condition (Figure 2D). Moreover, red light also significantly promoted the content of soluble sugar and pectin of MCB compared with blue light (Figures 2I,J). The soluble sugar and soluble pectin contents of the red-light treatment increased by 299.48 and 217.71%, respectively.

### Red Light Changed Chloroplast Ultrastructure of *Mesona chinensis* Benth Leaves

**FIGURE 3:**
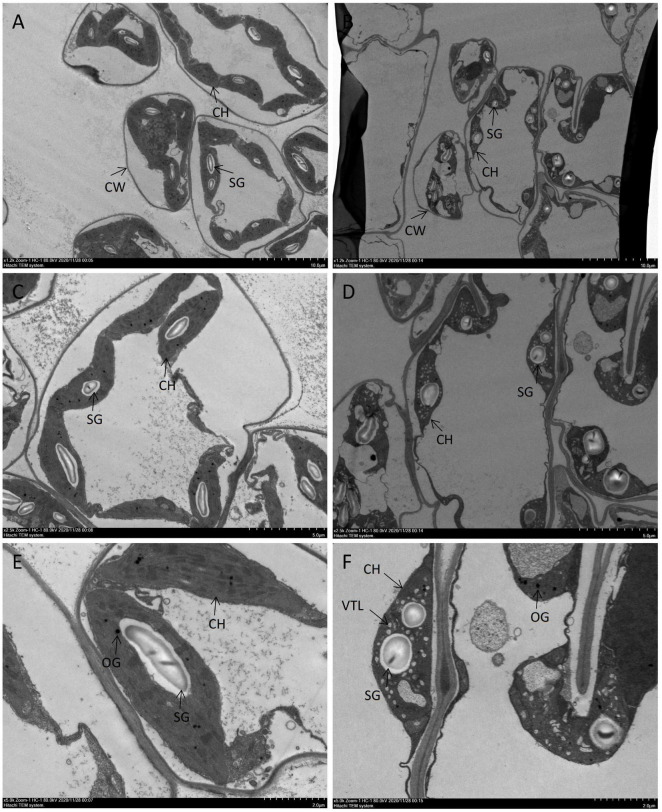
Comparison of the ultrastructure of MCB leaves under the red and blue lights. **(A,C,E)** Represented the blue-light treatment; **(B,D,F)** Represented the red-light treatment. SG, starchgrains; OG, osmiophilicgranules; CH, chloroplast; CW, cellwall; VTL, vesiculation of thylakoid lamellar.

As mentioned earlier, the leaves turned light yellow under the red-light treatment, while it was green under the blue-light treatment. To further study the effects of red and blue lights on the leaf ultrastructure of MCB, TEM observation was performed in this study (Figure 3). The leaves under both treatments had intact cell walls and chloroplast structures. The osmiophilic granules and starch grains were also observed in the leaves under both treatments. Compared with the blue-light treatment, there were more starch grains in the leaves under the red-light treatment. However, they had different chloroplast ultrastructures. Remarkably, a large number of vesicles were found and the vesiculation phenomenon was observed in thylakoid lamellae under the red-light treatment in comparison with the blue-light treatment.

### RNA Sequencing, *de novo* Assembly, and Functional Annotation

The RNA-Seq data generated in this study have been deposited in the Sequence Read Archive (SRA) database (accession number PRJNA741889). The *Q*30 values and the percentage of clean data of the six samples were more than 91 and 90%, respectively (Supplementary Tables 2, 3). A total of 171,484 transcripts and 60,064 unigenes were identified with a total length of 224,909,017 and 64,130,649 bp, respectively (Supplementary Table 4), and then the unigenes were annotated against NR, GO, KEGG, eggNOG, Swiss-Prot, and Pfam databases (Supplementary Tables 5, 6). Among these, 35,666 unigenes were annotated to the NR database, accounting for 59.38% of the transcripts, while 16,617 (27.67%), 14,347 (23.89%), 19,235 (32.02%), 34,247 (57.02%), and 26,555 (44.21%) unigenes could be annotated to GO, KEGG, Pfam, eggNOG, and Swissport, respectively (Supplementary Table 7). GO analysis revealed that a total of 24, 24, and 19 GO terms were involved in biological processes, cell components, and molecular functions, respectively (Supplementary Figure 1). Furthermore, we obtained the active biological functional pathways on MCB leaf unigenes from the KEGG pathway database. A total of 9,573 unigenes aligned with 35 classifications, and the pathways were divided into five categories containing metabolism, genetic information processing, environmental information processing, cellular processes, and organismal systems (Supplementary Figure 2).

### Identification of Differentially Expressed Genes and Pathways

**FIGURE 4:**
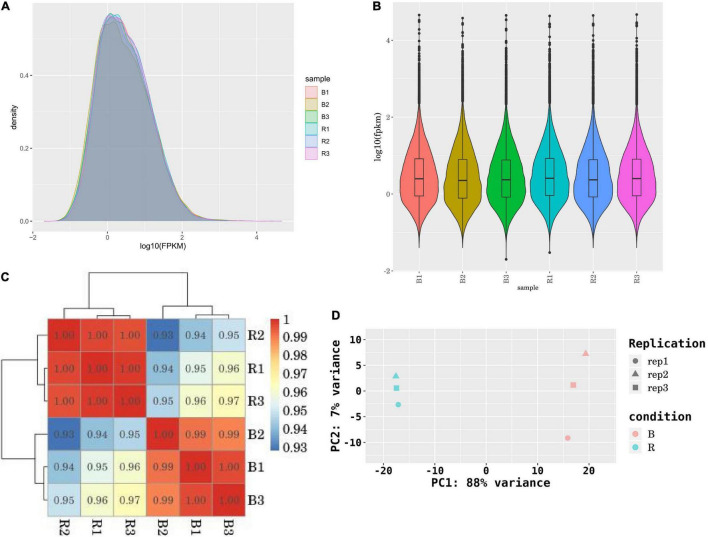
Analysis of RNA sequencing data of six samples. **(A,B)** The density distribution of FPKM; **(C)** Correlation test of six samples; **(D)** PCA analysis of six samples.

Using RSEM software and the transcript sequences as a reference, we aligned the clean reads of each sample to the reference sequence. Then, the number of reads aligned on each gene were counted in each sample and the FPKM values of each gene were calculated (Supplementary Table 8). The FPKM value between 1 and 10 was dominant in different ranges of expression levels (Figures 4A,B). Before DEGs analysis, the correlation of gene expression level among the samples was analyzed for checking the reliability of the experiment and the rationality of sample collection. The Pearson’s correlation coefficient of gene expression levels under the blue-light condition ranged from 0.93 to 0.97, while under the red-light condition it ranged from 0.99 to 1.00 (Figure 4C). In addition, the samples under the two treatments also differed remarkably by the principal component analysis (PCA) (Figure 4D). Therefore, it was indicated that the data could be used for further DEG analysis.

**FIGURE 5:**
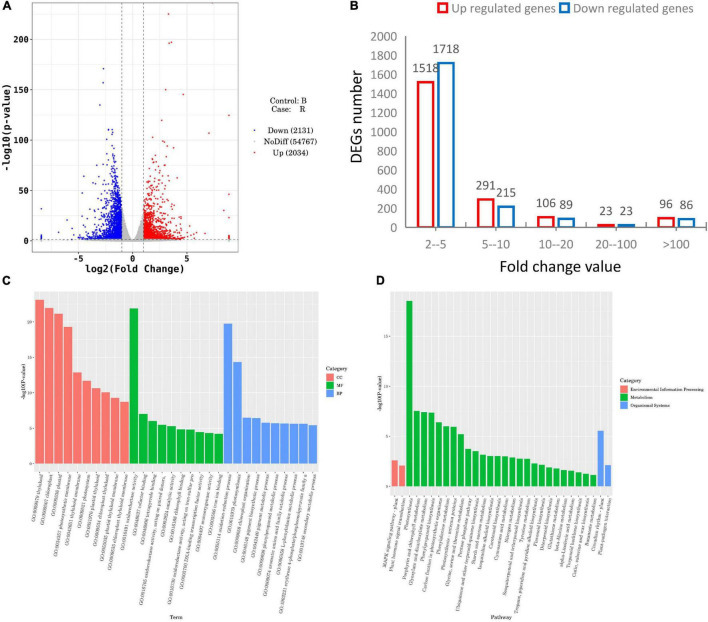
Differentially expressed genes (DEGs) statistics and functional enrichment analysis between the red- and blue-light treatments. **(A)** DEGs statistical analysis; **(B)** Distribution of DEGs based on different fold change thresholds; **(C,D)** GO and KEGG enrichment analysis, respectively.

To identify the genes involved in MCB growth, we analyzed the DEGs between the red-light and blue-light treatments with the following parameters: *p-*value < 0.05 and | log 2 FC| ≥ 1. A total of 4,165 DEGs were detected including 2,034 upregulated and 2,131 downregulated (Figure 5A). Among these, 2–5 fold changes were noted in the expression of majority DEGs (1,518 upregulated and 1,718 downregulated) (Figure 5B).

GO analysis unveiled that the DEGs were categorized into certain cellular components, molecular functions, and biological processes (Figure 5C). Cellular component analysis showed that the most significant enrichment of DEGs was involved in thylakoid, followed by chloroplast, plastid, and photosynthetic membrane. Regarding molecular functions, oxidoreductase activity was the most significant enrichment. In terms of biological processes, the oxidation-reduction process and photosynthesis were the significantly overrepresented items.

Further KEGG analysis uncovered that in total, 1,112 DEGs, including 410 upregulated and 702 downregulated genes, were associated with 111 pathways (Supplementary Table 9). All the top 30 most significant enrichment pathways were divided into environmental information processing, metabolism, and organismal systems (Figure 5D). Of these, only plant MAPK signaling pathways and plant hormone signal transduction were the most significant enrichment in environmental information processing, and the plant circadian rhythm and plant-pathogen interaction pathways were the two most representative pathways in organismal systems. Notably, the remaining 26 pathways, including starch and sucrose metabolism, pentose phosphate pathway, flavonoid biosynthesis, photosynthesis, and porphyrin and chlorophyll metabolism, were involved in metabolism.

Transcription factors regulate plant growth and development, environmental stress response, and biosynthesis of secondary metabolites by inhibiting or activating gene expression (Latchman, 1993; Chen et al., 2021). In this study, a total of 8,723 differentially expressed TFs were identified and they were distributed in 56 gene families (Supplementary Figure 3 and Supplementary Table 10). It was indicated that these TFs might be associated with MCB growth.

### Metabolome Profiling Between the Red- and Blue-Light Treatments

In this study, metabolites were extracted from leaf samples with six replicates and analyzed using LC-MS. A total of 184 metabolites were identified in the red- and blue-light treatments (Supplementary Table 11 and Supplementary Figure 4). Based on these metabolites, the PCA and relative standard deviation (RSD) showed that the data were reliable (Supplementary Figure 5). The metabolites included carbohydrates and carbohydrate conjugates (CCC), alcohols and polyols (AP), amino acids, peptides, and analogs (AAPA), fatty acids and conjugates (FAC), amines (A), eicosanoids (E), linoleic acids, and derivatives (LAD), 1-hydroxy-2-unsubstituted benzenoids (1H2UB), short-chain keto acids and derivatives (SKAD), tricarboxylic acids and derivatives (TAD), and cyclic purine nucleotides (CPN), accounting for 18.45, 19.42, 26.21, 11.65, 5.83, 3.88, 3.88, 2.91, 2.91, 2.91, and 1.94%, respectively (Supplementary Figure 6).

**FIGURE 6:**
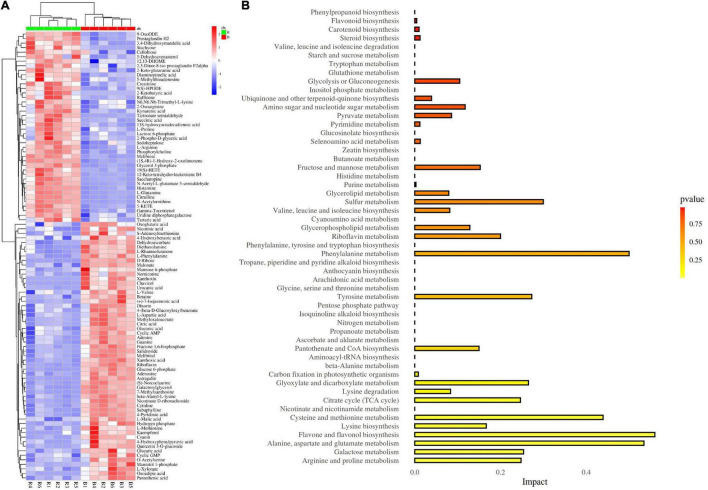
Analysis of differentially expressed metabolites (DEMs) and KEGG pathway. **(A)** DEMs identified between the red- and blue-light treatments; **(B)** Analysis of KEGG pathway of DEMs.

Furthermore, we found 99 DEMs between the red- and blue-light treatments, including 42 upregulated and 57 downregulated (Figure 6A). To illustrate the function of the metabolites involved in MCB growth, we analyzed the 99 DEMs using the KEGG database. A total of 53 pathways were found when the DEMs between the two treatments were introduced into KEGG (Figure 6B). Of these, based on the pathway impact scores (>0.1), we identified the 17 most relevant metabolic pathways (Table 1). Furthermore, seven pathways were at an extremely significant level (*p* < 0.01), including flavone and flavonol biosynthesis (FFB), aspartate and glutamate metabolism (AAGM), cysteine and methionine metabolism (CMM), galactose metabolism (GM), arginine and proline metabolism (APM), citrate cycle (TCA cycle), and lysine biosynthesis (LB). Only one pathway, glyoxylate and dicarboxylate metabolism, was at a significant level (*p* < 0.05). The remaining nine pathways were statistically non-significant (*p* > 0.05).

**TABLE 1:** Results from KEGG pathway analysis.

Pathway name	Total	Hits	Raw p	-Log(p)	Holm adjust	FDR	Impact
Flavone and flavonol biosynthesis	9	3	0.0263	3.6375	1.0000	0.5724	0.5600
Alanine, aspartate, and glutamate metabolism	22	5	0.0223	3.8038	1.0000	0.5724	0.5345
Phenylalanine metabolism	8	1	0.4736	0.7475	1.0000	1.0000	0.5000
Cysteine and methionine metabolism	34	6	0.0408	3.2001	1.0000	0.5910	0.4391
Sulfur metabolism	12	1	0.6187	0.4802	1.0000	1.0000	0.3000
Tyrosine metabolism	18	2	0.4085	0.8952	1.0000	1.0000	0.2727
Glyoxylate and dicarboxylate metabolism	17	3	0.1365	1.9912	1.0000	1.0000	0.2653
Galactose metabolism	26	6	0.0115	4.4636	0.9908	0.5012	0.2539
Arginine and proline metabolism	38	8	0.0065	5.0396	0.5635	0.5012	0.2480
Citrate cycle (TCA cycle)	20	4	0.0612	2.7936	1.0000	0.6656	0.2467
Riboflavin metabolism	10	1	0.5519	0.5944	1.0000	1.0000	0.2000
Lysine biosynthesis	10	3	0.0355	3.3373	1.0000	0.5910	0.1667
Fructose and mannose metabolism	16	1	0.7240	0.3229	1.0000	1.0000	0.1525
Pantothenate and CoA biosynthesis	14	2	0.2932	1.2270	1.0000	1.0000	0.1500
Glycerophospholipid metabolism	25	2	0.5852	0.5359	1.0000	1.0000	0.1283
Amino sugar and nucleotide sugar metabolism	41	2	0.8383	0.1764	1.0000	1.0000	0.1177
Glycolysis or Gluconeogenesis	25	1	0.8672	0.1425	1.0000	1.0000	0.1048

### Integrative Analysis of Transcriptome and Metabolome

**FIGURE 7:**
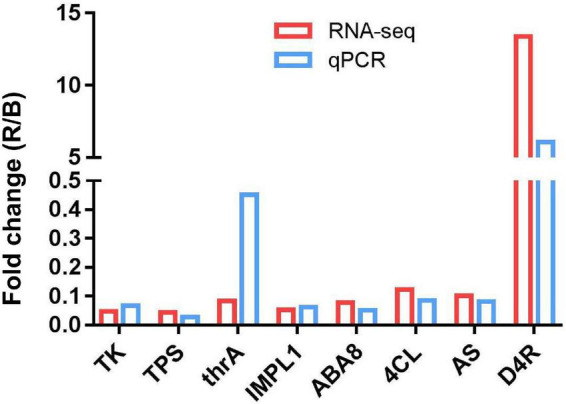
The expression level of *AS*, *TK*, *TPS*, *IMPL1*, *D4R*, *4CL6, thrA*, and *ABA8* between the red- and blue-light treatments.

Based on the DEGs and DEMs data, we conducted an integrative analysis of transcriptome and metabolome between the red- and blue-light treatments. Results showed that a total of 24 pathways included 70 compounds (metabolites) and were involved in 28 unigenes (Table 2). These pathways included starch and sucrose metabolism (C00092 and C00185), phenylpropanoid biosynthesis (C00079), cysteine and methionine metabolism (C00019, C00049, C00073, C00109, C00170, C00979), glycolysis/gluconeogenesis (C00631), and pentose and glucuronate interconversions (C00026 and C05411). These genes included *asparagine synthetase* (*AS*), *thymidine kinase* (*TK*), *alpha, alpha-trehalose-phosphate synthase* (*TPS*), *phosphatase IMPL1* (*IMPL1*), *dihydroflavonol 4-reductase* (*D4R*), and *4-coumarate-CoA ligase-like 6* (*4CL6*), *bifunctional aspartokinase-homoserine dehydrogenase* (*thrA*), and *abscisic acid 8′-hydroxylase 2 isoform X1* (*ABA8* or *CYP707A2*), which were differentially expressed between the two treatments (Figure 7). It was indicated that these pathways and genes might play important roles in the growth and development of MCB.

**TABLE 2:** Results of integrative analysis of transcriptome and metabolome.

KEGG	Pathway description	Compounds_KO	Genes_ko
ath00960	Tropane, piperidine, and pyridine alkaloid biosynthesis	C00079;C00253;C06524;	K00276
ath00350	Tyrosine metabolism	C00042;C01179;C05580;C06046;	K00276
ath00240	Pyrimidine metabolism	C00064;C00383;C00475;	K00857
ath00592	alpha-Linolenic acid metabolism	C16317;	K08241
ath00650	Butanoate metabolism	C00026;C00042;	K01641
ath00500	Starch and sucrose metabolism	C00092;C00185;	K16055
ath00270	Cysteine and methionine metabolism	C00019;C00049;C00073;C00109;C00170;C00979;	K12524
ath00300	Lysine biosynthesis	C00026;C00049;C00322;C00449;C00666;	K12524
ath00230	Purine metabolism	C00064;C00147;C00212;C00242;C00575;C00942;	K00873
ath00562	Inositol phosphate metabolism	C00092;	K01092
ath00906	Carotenoid biosynthesis	C13453;C13454;	K09843
ath00950	Isoquinoline alkaloid biosynthesis	C01179;C06160;	K00276
ath00620	Pyruvate metabolism	C00149;	K00873
ath00360	Phenylalanine metabolism	C00042;C00079;C00156;	K00276;K10775
ath00010	Glycolysis/Gluconeogenesis	C00631;	K00873
ath00130	Ubiquinone and other terpenoid-quinone biosynthesis	C00156;C01179;C03993;	K01904
ath00250	Alanine, aspartate, and glutamate metabolism	C00026;C00042;C00049;C00064;C00940;	K01953
ath00410	beta-Alanine metabolism	C00049;C00383;C00864;C05341;	K00276
ath00941	Flavonoid biosynthesis	C05903;	K13082
ath00940	Phenylpropanoid biosynthesis	C00079;	K01904;K13066;K10775
ath00260	Glycine, serine and threonine metabolism	C00049;C00109;C00631;C00719;	K12524;K00276
ath00280	Valine, leucine, and isoleucine degradation	C00183;	K01641
ath00970	Aminoacyl-tRNA biosynthesis	C00049;C00062;C00064;C00073;C00079;C00148;C00183;	K04567
ath00040	Pentose and glucuronate interconversions	C00026;C05411;	K01051

### Verification of Differentially Expressed Genes Using Quantitative Reverse Transcription -PCR

To verify the credibility of transcriptome sequencing data, eight candidate DEGs (*AS*, *TK*, *TPS*, *IMPL1*, *D4R*, *4CL*, *thrA*, and *ABA8*) were selected and analyzed using qRT-PCR. Results showed that our data were in line with those obtained with the RNA-Seq (Figure 7). These indicated the reliability of the results of DEGs analysis.

## Discussion

### Red Light Promoted the Growth and Development and Quality of *Mesona chinensis* Benth

Light is the basic energy source of photosynthesis and the main environmental factor regulating plant growth and development throughout the plant life cycle (Devlin et al., 2007). The growth and development of plants are not only restricted by light intensity but also affected by light quality, that is, the light and radiation of different wavelengths (Paradiso and Proietti, 2021). The solar spectrum is roughly divided into ultraviolet radiation (ultraviolet, UV < 400 nm: UV-A, 320–400 nm; UV-B, 280–320 nm; UV-C, <280 nm, 100–280 nm), visible or photosynthetically active radiation (PAR) (PAR, 400–700 nm: blue light, 400–500 nm; green light, 500–600 nm; red light, 600–700 nm), and infrared radiation (700–800 nm) (Xu et al., 2015). Red light and blue light are the main energy sources for carbon dioxide assimilation and have primary impacts on carbohydrate biosynthesis and plant growth (Lim and Eom, 2013; He et al., 2020b). Red light affected the height and leaf area of kidney bean plants (Hiromichi and Kazuhiro, 2000) and potato plantlets (Miyashita et al., 1997; Lee et al., 2011). Bantis et al. (2016) reported that the red light increased the dry weight of watermelon seedlings. Peanut and lettuce plants under a high proportion of red light also exhibited enhanced biomass accumulation (Poulet et al., 2014; Li et al., 2018). Red light determined better growth compared with blue light in lettuce (Yanagi et al., 1996). In this study, red light promoted the growth and development of MCB in comparison with blue light, specifically in the plant height, dry and fresh weight, and root growth (Figures 2A–H). It was consistent with the results mentioned earlier. The difference was that the red light reduced chlorophyll content in leaves of MCB compared with the blue light. It was consistent with the results of the study on Welsh onion (Gao et al., 2020). The reason might be that the chlorophyll content could be increased by the blue light (He et al., 2020b).

In response to the alterations in the light spectrum, plants are capable of adapting to environmental changes by accumulating a variety of metabolites, including polysaccharides, flavonoids, triterpenoids, and phenolic compounds (Ibrahim and Jaafar, 2012). Studies reported that the red light increased the number of phenolic compounds in the leaves of lettuce and tomato stems (Li and Kubota, 2009; Kim et al., 2013), *Ocimum basilicum* (Bantis et al., 2016), and *Perovskia* lamiaceae (Ghaffari et al., 2019), and it also promoted the anthocyanin content in *Brassica oleracea* L. *var*. acephala D.C. (Lefsrud et al., 2008) and red cabbage leaves (Mizuno et al., 2001). Meanwhile, under the red light, the contents of soluble sugar and total sugar significantly increased in tomatoes (Pu et al., 2005). In this study, the red light significantly promoted the contents of soluble sugar and pectin of MCB compared with the blue light (Figures 2I,J). Pectin was an important component of MCB polysaccharides, which was the standard to measure the quality of MCB. As the red light had positive effects on the biomass and quality of MCB, it might be feasible to supplement red light in production to promote the growth and development and quality of MCB.

### Responses of Chloroplast Ultrastructure of *Mesona chinensis* Benth Leaves to the Red and Blue Lights

Chloroplasts contain chlorophyll and are rich in thylakoid membranes that can absorb and transform light energy (Kirchhoff, 2019) so that they are the sites of photosynthesis in plant cells (Barry et al., 2012; Tang et al., 2018). If the chlorophyll synthesis was decreased or impeded, the chloroplast ultrastructure would change (Zhang et al., 2014). The light quality was one of the important factors affecting chloroplast development. Under the blue light, the number of grana lamellae was the highest with the most stacked lamellae and the minimum starch grains in the chloroplast, while the leaves developed under red light alone displayed dysfunctional photosynthetic apparatus (Wang et al., 2015). In upland cotton, the seedlings that were grown under blue LEDs also showed high integrity of the chloroplast ultrastructure with a visible lamellar structure (Li et al., 2010). Gao et al. (2020) reported that the chloroplasts of leaves treated with blue and red lights were intact and contributed to photosynthesis, while yellow light inhibited chloroplast development. In our investigation, the leaves under both the red and blue light treatments also had intact chloroplast ultrastructure. However, compared with the blue-light treatment, there were more starch grains in the leaves under the red-light treatment, and a large number of vesicles were found in the thylakoid lamellar of the leaves under the red-light treatment (Figure 3). It could be concluded that the blue light was a key signal for chloroplast development (Wang et al., 2015). However, the red light had different effects on chloroplast development. These comparisons could support the hypothesis that there were species-specific responses to the light environment (Gao et al., 2020).

### Contributing to Understanding the Chemical Components of *Mesona chinensis* Benth

Previous studies showed that MCB contained polysaccharides, flavonoids, triterpenoids, phenols, and other chemical components (Lin et al., 2013). MCP consisted of eight monosaccharides, including mannose, rhamnose, ribose, glucuronic acid, galacturonic acid, glucose, galactose, and xylose (Zhang et al., 2013). Quercetin was the main component of flavonoids (Liu, 1995), ursolic acid and oleanolic acid were the predominant components of triterpenoids (Shyu et al., 2008), and caffeic acid (the highest content) and epicatechin were the primary components of phenols (Qiu et al., 2010) in MCB. Besides the polysaccharides, flavonoids, triterpenoids, and phenols, MCB also contained minerals (such as iron, calcium, magnesium, manganese, zinc, and potassium) (Lin and Zhu, 1992), vitamin B, amino acids, cellulose, and plant pigments, etc. (Liu and Chen, 2004; Cao et al., 2007; Qiu et al., 2009). In this study, we identified 184 metabolites in MCB (Supplementary Table 11 and Supplementary Figure 4), which positively contributed to understanding the chemical components of MCB and laid a foundation for the future study of chemical components in MCB.

### Metabolites Involved in the Growth and Development of *Mesona chinensis* Benth

In this study, a total of 99 DEMs (42 upregulated and 57 downregulated) were found between the red- and blue-light treatments (Figure 6A). Furthermore, based on the KEGG analysis, seven pathways, including FFB, AAGM, CMM, GM, APM, TCA cycle, and LB, were at an extremely significant level (*p* < 0.01), and only the glyoxylate and dicarboxylate metabolism pathway was at a significant level (*p* < 0.05) (Table 1). Therefore, it was indicated that these pathways might be involved in the growth and development of MCB.

### Genes Involved in the Growth and Development of *Mesona chinensis* Benth

Our integrative analysis of transcriptome and metabolome unveiled that 28 DEGs included *AS*, *TK*, *TPS*, *IMPL1*, *D4R*, and *4CL6*, *thrA*, and *ABA8* or *CYP707A2*. Asparagine (also known as aspartamide) was α-amino acid that was particularly found in plant proteins. Asparagine possessed a high nitrogen-to-carbon ratio and was the predominant nitrogen transport compound utilized when carbon sources were relatively limited in the dark (Sieciechowicz et al., 1988). The *AS* genes appeared to be encoded by a small gene family in most plant species, such as *Arabidopsis* (Lam et al., 1998), sunflower (Herrera-Rodriguez et al., 2002), *Triticum aestivum* (Gao et al., 2016), and *H. vulgare* (Avila-Ospina et al., 2015), and the *AS* gene expression in higher plants was regulated by many factors, for example, light, organ type, and development. Tsai and Coruzzi (1990) identified a family of genes (*AS1* and *AS2*) in *Pisum sativum*, and the *AS* genes were preferentially expressed in plants grown in the dark; moreover, the mRNA of the *AS* genes was negatively regulated by light at the transcriptional level and the expression of *AS* genes fluctuated sharply during a “normal” light/dark cycle. Wang et al. (2005) demonstrated that the *TaAsnS1* expression in bread wheat seedlings was significantly induced by osmotic and salinity stresses, probably through ABA-dependent pathways. *AsnS1* genes were downregulated in *N*-stressed roots, stems, and leaves during seedling growth and booting, while *AsnS2* genes were expressed in leaves, stems, and roots (Curci et al., 2018). In our investigation, the *AS* gene was differentially expressed between the leaves under the red- and blue-light conditions. The negative regulation of the *AS* gene expression by light was shown to be a general phenomenon in plants, which also occurs in non-legumes such as *Nicotiana tabacum* and *Nicotiana plumbaginifolia* (Tsai and Coruzzi, 1991).

Thymidine kinase (TK) catalyzed the first step by transferring a phosphate group to a thymidine molecule in the nucleotide salvage pathway. In *Oryza sativa*, the *TK1* gene expression was independent of cell-cycle regulation as the transcript was present in all developmental stages, and it was even more abundant in non-proliferating tissues (Ullah et al., 1999). In *Hevea brasiliensis*, the rubber tree, upregulation of the *TK1* gene was closely associated with resistance to mechanical wounding (Venkatachalam et al., 2010). There were two thymidine kinase genes, *AtTK1a* and *AtTK1b*, in *Arabidopsis thaliana*. *TK1a* was expressed in most tissues during plant development, and it was differentially induced by ultraviolet-C radiation because *TK1b* expression was unaffected (Pedroza-García et al., 2014). While mutants for each *TK1* gene showed normal growth, the double mutant developed poorly and plantlets died at an early stage, indicating that the function of *TK1* was essential for plant development (Clausen et al., 2012).

Myo-inositol was a key precursor of various phosphate metabolites in eukaryotes, for example, cell wall polysaccharides, phosphatidylinositol, phytic acid, and indole-3-acetic acid conjugate of myo-inositol (Loewus and Murthy, 2000). Myo-inositol monophosphatase (IMP) catalyzed the dephosphorylation of myo-inositol 3-phosphate in the last step of myo-inositol biosynthesis, which was also important in phosphate metabolism and was required for the biosynthesis of phytic acid, cell wall polysaccharides, and phosphatidylinositol. IMP was encoded by *VTC*4; however, *IMPL1* and *IMPL2* were the two additional and putative *IMP* genes in *A. thaliana* (Torabinejad et al., 2009). Sato et al. (2011) demonstrated that the loss-of-function mutant *impl2* leads to embryonic lethality at the globular stage, and *IMPL2* was also involved in histidine biosynthesis during embryo development. In developing seeds of *A. thaliana*, the expression of *IMP* genes was not coupled with the expression of the genes encoding myo-inositol phosphate synthases, which supplied the substrate for IMPs, but was correlated with the expression of the gene for myoinositol polyphosphate 1-phosphatase (SAL1), which was involved in the myo-inositol salvage pathway, indicating a possible salvage pathway role in the seed development (Sato et al., 2011).

Trehalose metabolism was ubiquitous in plants, and the genes encoding trehalose pathway constituents were first reported in *A. thaliana* (Vogel et al., 1998). There were 11 trehalose phosphate synthase (TPS) homologs in *A. thaliana*. In particular, the *TPS* genes were expressed at very low levels (Schluepmann et al., 2003), and the *AtTPS1* gene was expressed in all tissues and was essential during embryogenesis (Eastmond et al., 2002), indicating an important role for trehalose metabolism in plants. *OtsA* encoded a TPS, and the expression of *OtsA* accumulated trehalose 6-phosphate (T6P). Moreover, the plant phenotype with T6P accumulation was significantly opposite to that of plants with low T6P levels and was consistent with the key role of T6P in growth and development (Schluepmann et al., 2003).

In addition, *D4R*, catalyzing the reduction of dihydroflavonols to leucoanthocyanins, was a key enzyme in the biosynthesis of anthocyanidins, proanthocyanidins, and other flavonoids, which was of great significance for plant development (Li et al., 2012). In *A. thaliana*, two 4-coumarate- CoA ligase (4CL)-like proteins (*At4g05160* and *At5g63380*) were targeted to leaf peroxisomes and could contribute to jasmonic acid biosynthesis (Schneider et al., 2005), which was a plant-signaling molecule closely associated with plant resistance to abiotic stress (Wang et al., 2020). In *Escherichia coli*, *thrA* catalyzes the commitment step involved in the regulation of the biosynthesis of threonine (Angeles and Viola, 1990), which can improve plant tolerance and promote the process of humification. Abscisic acid (ABA) is a plant stress hormone, and *ABA 8′-hydroxylase* (CYP707A) is the major and key P450 enzyme in ABA catabolism in plants (Ueno et al., 2007).

Taken together, in this study, compared with the blue-light treatment, the *AS*, *TK*, *TPS*, *IMPL1*, *4CL*, *thrA*, and *ABA8* genes were downregulated, while the *D4R* gene was upregulated under the red-light condition (Figure 7). The expression of these genes from the leaves of MCB could be regulated by light quality, indicating that these genes might be closely related to the growth and development of MCB.

## Conclusion

**FIGURE 8:**
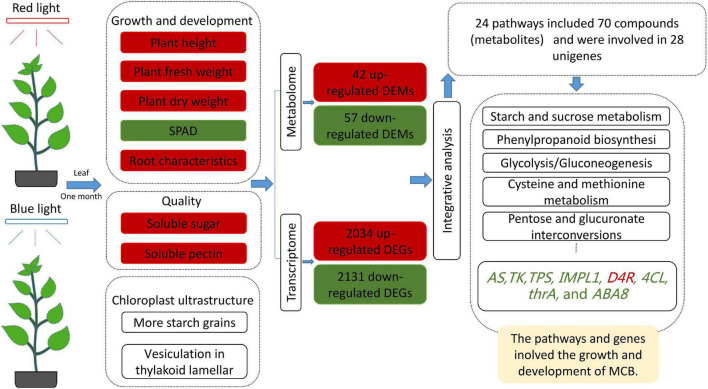
Overview of the red light and blue light regulating the growth and development of MCB. Red boxes and words indicated upregulation; Green boxes and words indicated downregulation.

The red light promoted the growth and development and quality of MCB in comparison with the blue light. The plant phenotype and leaf chloroplast ultrastructure responded differently to the red and blue lights. Transcriptome analysis showed 410 upregulated and 702 downregulated unigenes. The results of metabonomics revealed that a total of 184 metabolites and 99 DEMs were identified between the red- and blue-light treatments. Integrative analysis of transcriptome and metabolome unveiled that *AS*, *TK*, *TPS*, *IMPL1*, *4CL*, *D4R*, *thrA*, and *ABA8* genes were differentially expressed (Figure 8). Therefore, these pathways and genes might be involved in the growth and development of MCB.

## Data Availability Statement

“The original contributions presented in the study are publicly available. This data can be found here: National Center for Biotechnology Information (NCBI) BioProject database under accession number PRJNA741889.”

## Author Contributions

DT was involved in conceptualization, methodology, investigation, formal analysis, writing original draft, and writing—reviewing and editing. FW and JM were involved in funding acquisition. FW, QH, KW, and XY were involved in writing—reviewing and editing. All authors approved the submitted version.

## Conflict of Interest

The authors declare that the research was conducted in the absence of any commercial or financial relationships that could be construed as a potential conflict of interest.

## Publisher’s Note

All claims expressed in this article are solely those of the authors and do not necessarily represent those of their affiliated organizations, or those of the publisher, the editors and the reviewers. Any product that may be evaluated in this article, or claim that may be made by its manufacturer, is not guaranteed or endorsed by the publisher.

## Footnotes

1 http://trinityrnaseq.sourceforge.net/, version 2.8.5.

2 ftp://ftp.ncbi.nlm.nih.gov/blast/db/, version v0.8.37.99.

3 http://www.genome.jp/kegg/, version 2.1.1.

4 http://www.geneontology.org/, version 2.5.0.

5 http://www.ncbi.nlm.nih.gov/COG/, version v0.8.37.99.

6 http://web.expasy.org/docs/swiss-prot_guideline.html, version v0.8.37.99.

7
www.bioconductor.org

8
http://metlin.scripps.edu

9
http://www.hmdb.ca

10
http://www.massbank.jp/

11
https://www.mzcloud.org

12
http://www.lipidmaps.org
