# Supplementary material for: Identification of Differentially Expressed Genes and Pathways Involved in Growth and Development of Mesona chinensis Benth Under Red- and Blue-Light Conditions
Source: Front Plant Sci. 2021 Nov 25;12:761068. doi: 10.3389/fpls.2021.761068 (PMC8656965; doi:10.3389/fpls.2021.761068)
Supplement: Supplementary file 1 [file Data_Sheet_1.DOCX]

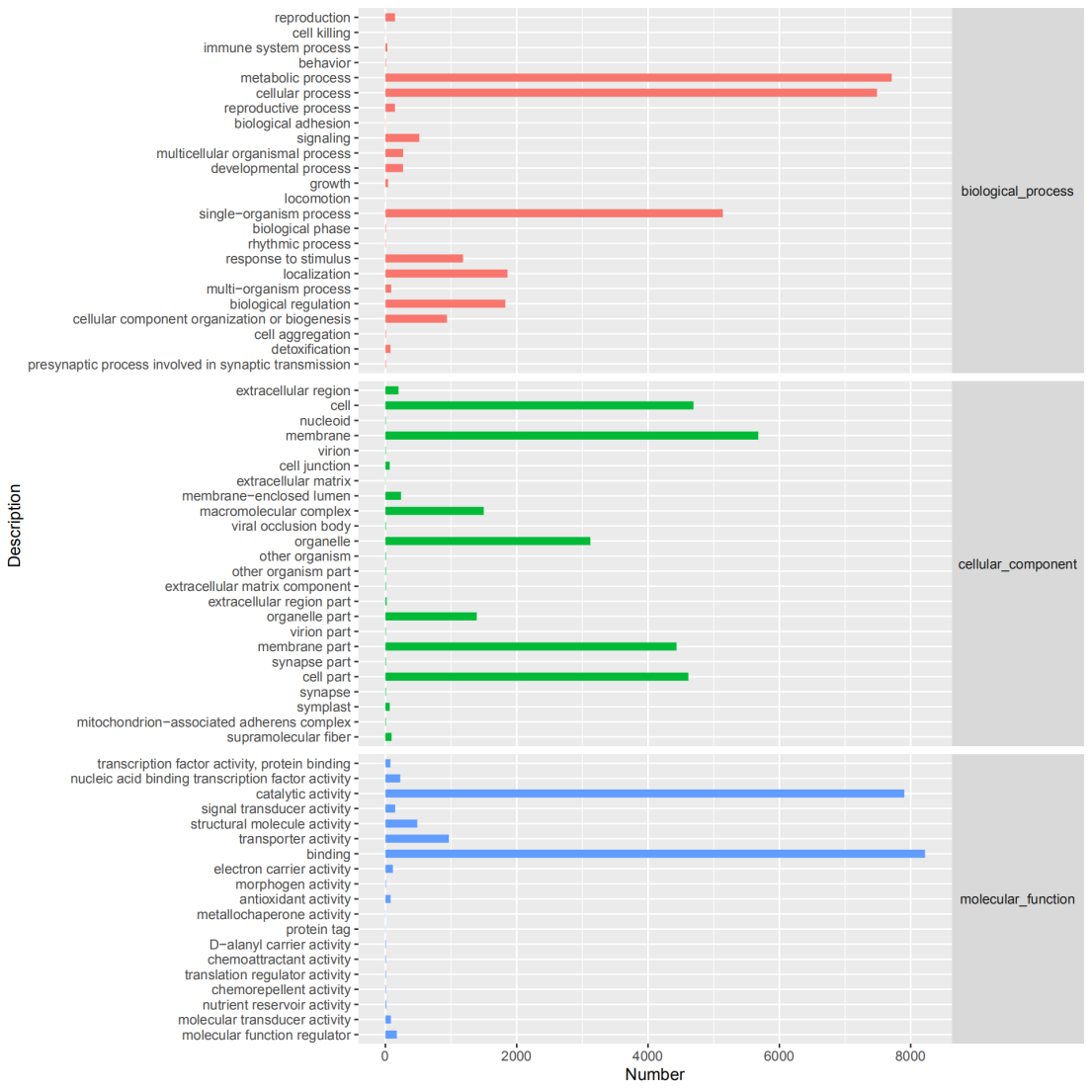


**Fig. S1** GO annotation statistics.


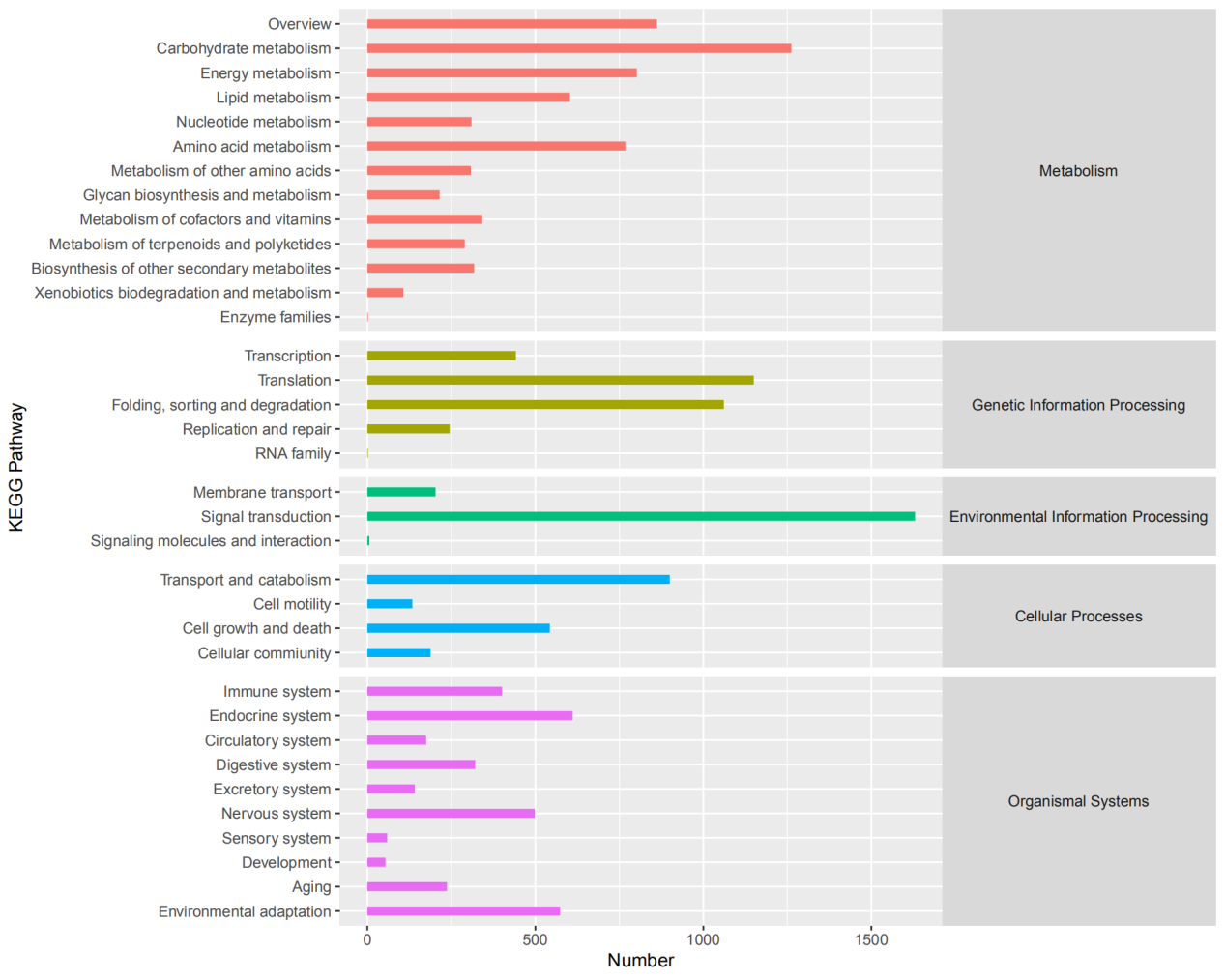


**Fig. S2** KEGG annotation statistics.


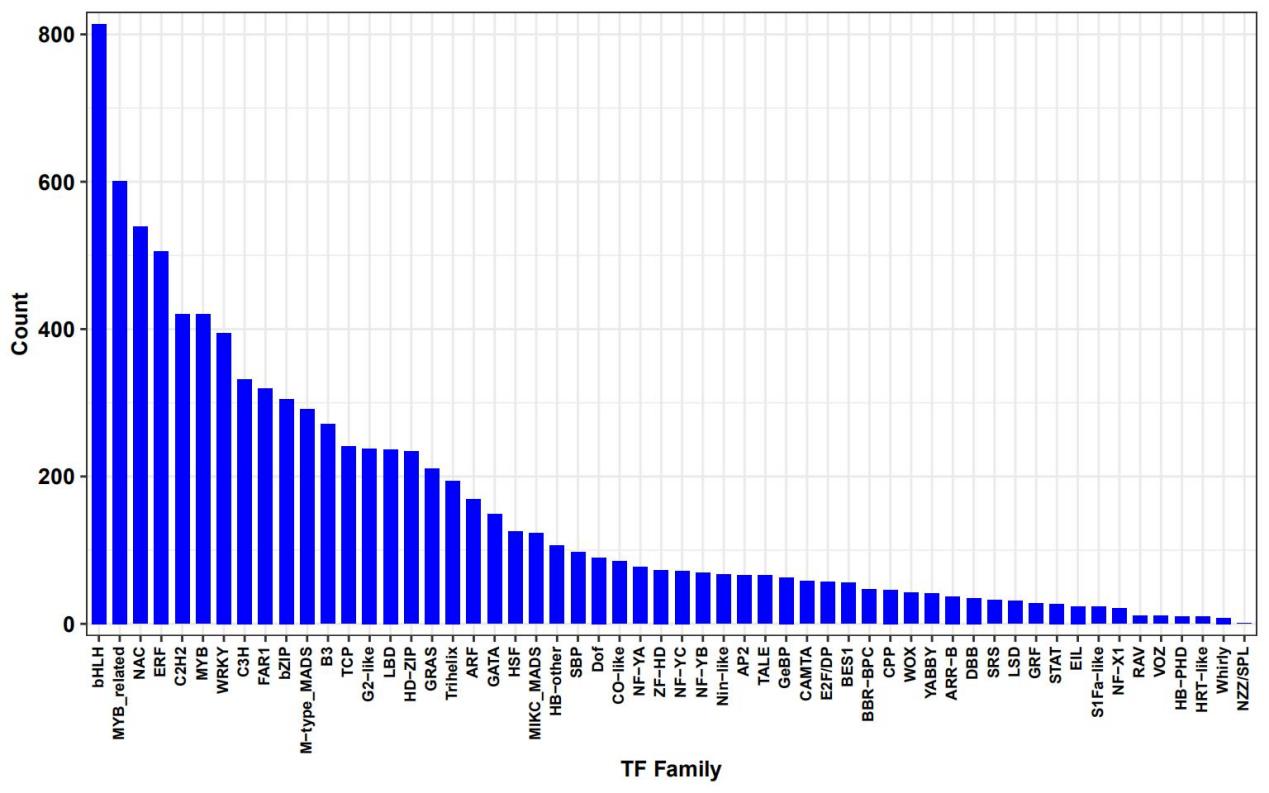


**Fig. S3** Differentially expressed TFs between the red and blue light treatments.


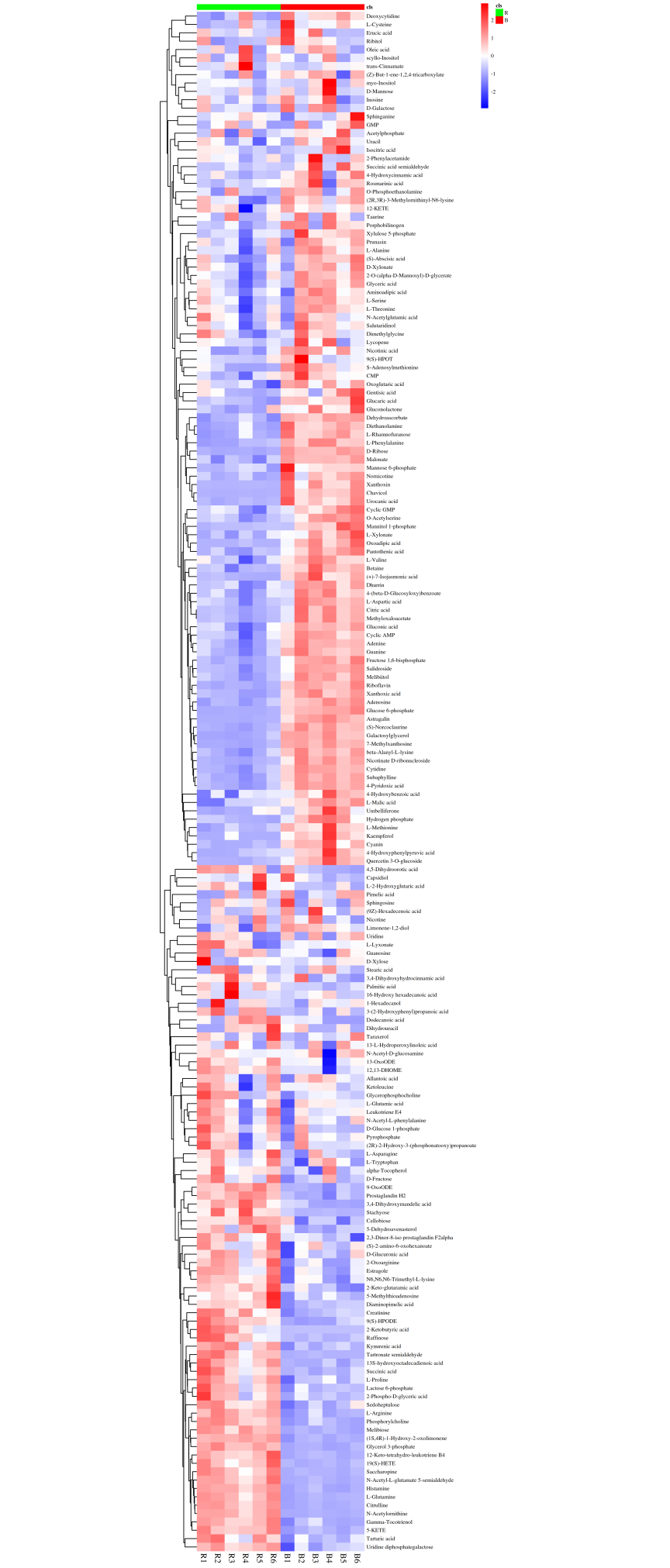


**Fig. S4** The 184 metabolites were identified in red and blue light treatments.


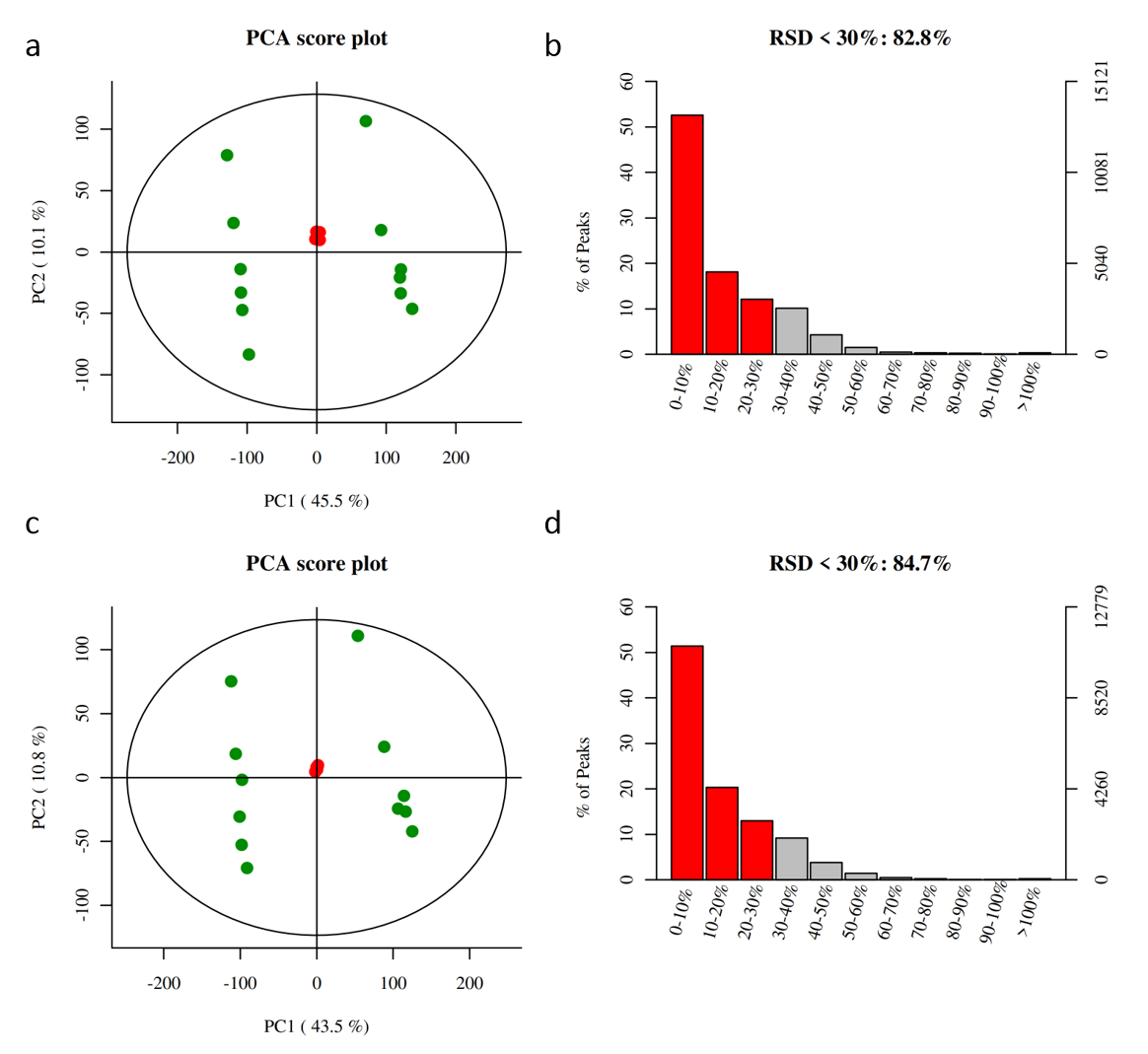


**Fig. S5** Quality control and quality assurance of the samples. a, b, positive ion mode; c, d, negative ion mode; a, c, PCA score plot; b, e: analysis of relative standard deviation, RSD.


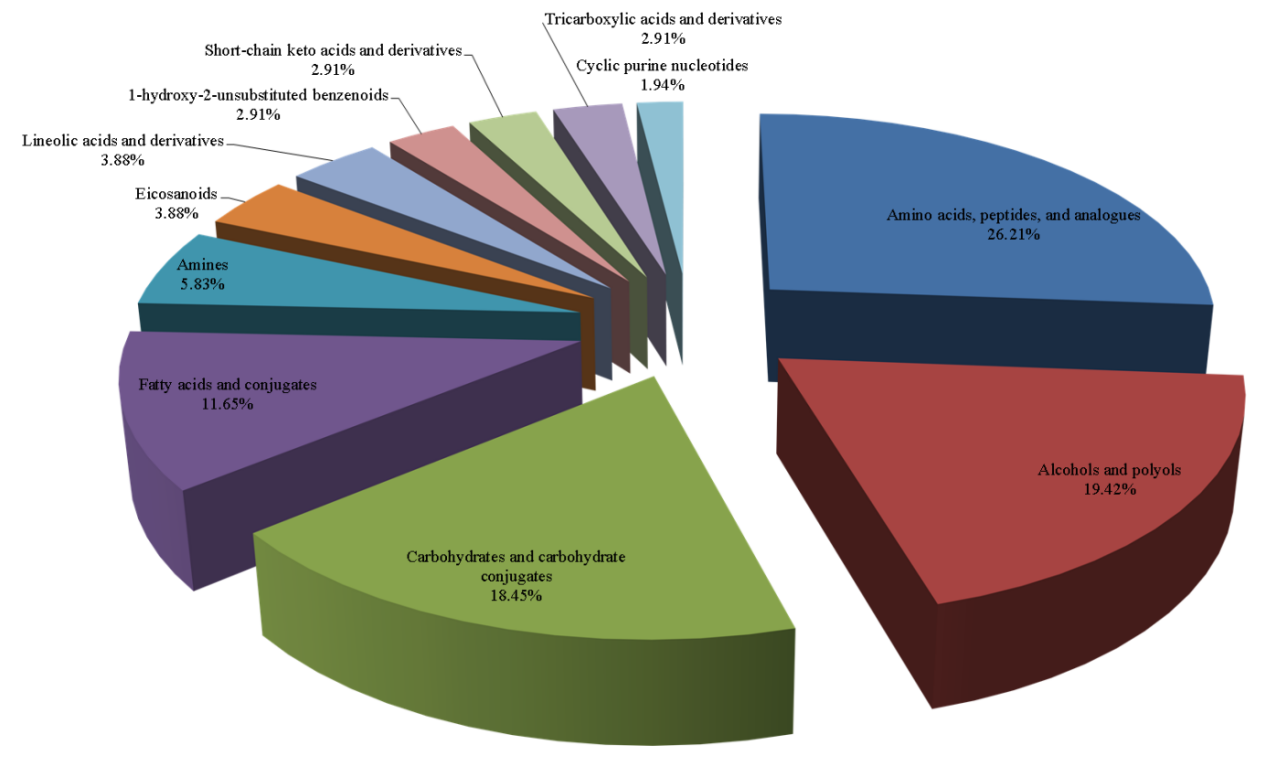


**Fig. S6** The classification, description and and statistics of all the metabolites identified in MCB leaves under red and blue light conditions. a, b, the classification and and statistics of metabolites; c, chemical structure classification of metabolites; d, subclass classification of chemical structure of metabolites.
